# Drought stress promotes the colonization success of a herbivorous mite that manipulates plant defenses

**DOI:** 10.1007/s10493-017-0200-4

**Published:** 2017-11-29

**Authors:** Miguel G. Ximénez-Embún, Joris J. Glas, Felix Ortego, Juan M. Alba, Pedro Castañera, Merijn R. Kant

**Affiliations:** 1 Department of Environmental Biology, Centro de Investigaciones Biológicas, CSIC, Madrid, Spain; 2 Institute for Biodiversity and Ecosystem Dynamics, University of Amsterdam, Amsterdam, The Netherlands

**Keywords:** Abiotic stress, *Aculops lycopersici*, Climate change, Herbivory, Hormones, Intermediary metabolism, Tomato russet mite

## Abstract

Climate change is expected to bring longer periods of drought and this may affect the plant’s ability to resist pests. We assessed if water deficit affects the tomato russet mite (TRM; *Aculops lycopersici*), a key tomato-pest. TRM thrives on tomato by suppressing the plant’s jamonate defenses while these defenses typically are modulated by drought stress. We observed that the TRM population grows faster and causes more damage on drought-stressed plants. To explain this observation we measured several nutrients, phytohormones, defense-gene expression and the activity of defensive proteins in plants with or without drought stress or TRM. TRM increased the levels of total protein and several free amino acids. It also promoted the SA-response and upregulated the accumulation of jasmonates but down-regulated the downstream marker genes while promoting the activity of cysteine—but not serine—protease inhibitors, polyphenol oxidase and of peroxidase (POD). Drought stress, in turn, retained the down regulation of JA-marker genes and reduced the activity of serine protease inhibitors and POD, and altered the levels of some free-amino acids. When combined, drought stress antagonized the accumulation of POD and JA by TRM and synergized accumulation of free sugars and SA. Our data show that drought stress interacts with pest-induced primary and secondary metabolic changes and promotes pest performance.

**Electronic supplementary material:**

The online version of this article (10.1007/s10493-017-0200-4) contains supplementary material, which is available to authorized users.

## Introduction

Global agriculture faces a big challenge as climate change will affect crop production in the near future. According to the Intergovernmental Panel on Climate Change (IPCC 2013), temperatures will increase and there will be more periods of drought, especially in semiarid zones. Drought is considered the main environmental factor limiting plant growth and yield worldwide (Chaves et al. 2003). Drought conditions are often associated with herbivore outbreaks (Mattson and Haack 1987), but both positive and negative effects on herbivores have been reported depending on the severity of the stress and differing across species and across the plants they are attacking (Huberty and Denno 2004; Cornelissen 2011; White 2009; Gutbrodt et al. 2011). Yet, plant responses to a combination of abiotic (e.g., drought stress) and biotic (e.g., herbivory) stresses and its impact on the performance of mite pests are poorly documented (Huberty and Denno 2004).

When a plant detects drought, it activates a series of tolerance mechanisms. First it will close the stomata but if the stress continues it will stop growing while it may reset its metabolism (Harb et al. 2010; Hummel et al. 2010). In order to prevent desiccation, cells undergo an osmotic adjustment, increasing the amount of free sugars and free amino acids, especially proline (Hummel et al. 2010; Showler 2013). These metabolic changes in the plant, have as consequence an increase on plant nutritional value for herbivores and therefore can promote their performance (Huberty and Denno 2004; White 2009). However, herbivores such as mites appear not to be passive and may themselves manipulate the plant’s primary metabolism (Zhou et al. 2015) and secondary metabolism i.e., defenses (Kant et al. 2015) to their own benefit. Plant stress responses are regulated by a complex network of phytohormones, with jasmonic acid (JA) and salicylic acid (SA) as the central players assisted by ancillary hormones such as abscisic acid (ABA), auxins and ethylene. The response to spider mite feeding is predominantly regulated by the JA pathway (Li et al. 2002; Ament et al. 2004; Kant et al. 2004; Zhurov et al. 2014; Schimmel et al. 2017a, b) and in to lesser extend by the SA-pathway (Villarroel et al. 2016). The JA pathway generates the active component JA isoleucine (JA-Ile) via oxophytodienoic acid (OPDA) and JA which initiates the expression of distinct defense genes via interaction with the proteasome (Schuman and Baldwin 2016). SA activates the expression of genes largely different from those activated by JA (Mur et al. 2006; De Vleesschauwer et al. 2014) and is the key regulator of defense responses induced by phloem feeding insects and biotrophic pathogens (Howe and Jander 2008). Why SA-signaling affects spider mites is unclear (Villarroel et al. 2016). Finally, drought stress gives rise to accumulation of ABA, which regulates processes like stomatal closure (Verma et al. 2016) which likely affects spider mites that prefer to feed via stomata (Bensoussan et al. 2016). Phytohormones can crosstalk and thereby modulate each other’s actions (Pieterse et al. 2009; Berens et al. 2017).

The tomato russet mite (TRM), *Aculops lycopersici* (Massee), is a cosmopolitan pest on solanaceous crops, mainly on tomato (*Solanum lycopersicum* L.). TRM causes massive yield losses of tomato (Duso et al. 2010), one of the most important horticultural crops worldwide. TRM is an eriophyoid mite, a family that includes the world’s smallest terrestrial animals (Keifer 1946; Sabelis and Bruin 1996) and, therefore, is often detected too late by growers. It has a short life completing its life-cycle within 7 days, depending on the temperature (Haque and Kawai 2003). They feed from epidermal cells by means of three sets of approximately 15 μm long stylets—derived from the chelicerae, the labrum and the infracapitulum, respectively—surrounding the mouth. These stylets are believed to be all inserted into the host: the cheliceral stylets probably deliver saliva into the epidermal cell while the infracapitular stylets and the labrum form a food channel to swallow the—possibly preorally digested—cell contents (Nuzzaci and Alberti 1996). TRM feeding destroys the upper and lower epidermal cells, among which guard cells, and induces formation of callous tissue appears in these regions while the plant suffers from strongly reduced photosynthesis and respiration (Royalty and Perring 1988). Once detected, TRM is difficult to control since it hides in the forest of tomato leaf hairs (trichomes) that protects it from predators (van Houten et al. 2013). Early studies on the TRM-tomato interaction reported that TRM induces accumulation of oxidative enzymes like peroxidases (POD) but not protease inhibitors (PI) or polyphenol oxidases (PPO) when feeding on the plant (Stout et al. 1996; Petanovic and Kielkiewicz 2010). In addition, Glas et al. (2014) described that TRM manipulates tomato plant defenses by suppressing JA-defenses, but not SA-defenses, downstream of phytohormone accumulation and independent from JA-SA antagonistic crosstalk. Together these effects were shown to promote competing spider mites on distal undamaged tissues (Glas et al. 2014) but possibly hamper these on a more local scale since they often depend on open stomata to reach the parenchyma (Bensoussan et al. 2016). TRM outbreaks might be promoted directly by climate change as it´s optimal growth conditions are at 27 °C and 30% relative humidity (Duso et al. 2010). However, we are primarily interested in how drought stress interacts with TRM-induced stress given that both response types affect similar hormonal pathways. TRM is particularly interesting because its affects hormonal signaling differently than the spider mites *T. urticae* and *T. evansi*. *Tetranychus urticae* induces both JA and SA responses (Kant et al. 2004; Alba et al. 2015). Tomato-adapted and non-adapted strains of *T. urticae* appeared to benefit from drought stress in tomato plants because of the improved nutritional value of the leaves (Ximénez-Embún et al. 2017). *Tetranychus evansi* was shown to suppress both JA and SA defenses simultaneously (Alba et al. 2015; Schimmel et al. 2017a, b), and its performance is promoted on plants under mild and moderate watering regimes probably due to increased levels in free sugars and essential amino acids. This indicates that indirect plant-mediated effects independent from defenses may promote population growth of this mite (Ximénez-Embún et al. 2016). Finally, TRM selectively suppresses only JA-defenses (Glas et al. 2014), whereas, in theory, a drought-induced increased in ABA could modulate JA-defenses beyond the control of the mite (Golldack et al. 2014).

The overall aim of this study was to assess the extent to which drought affects TRM-induced changes in the primary and secondary metabolism of plants and TRM performance. Moreover, we aimed to assess the extent to which their combination affects the physiological status (i.e., nutritional value and chemical defenses) of tomato in order to estimate the magnitude of the interaction between these two stresses. This information, together with the previously collected data on *T. urticae* and *T. evansi*, could proof to be a crucial instrument for predicting outbreaks of mite pests that affect plant resistance in distinct manners during a changing climate.

## Materials and methods

### Plant material and mite rearing

Tomato (cv. Moneymaker) seeds were germinated in soil (commercial peat) and transferred to 0.66-L pots (diameter: 12 cm) filled completely with the same soil and grown in a greenhouse with day/night temperatures of 23 to 18°C (day/night) and a 16/8 h light/dark regime.

A tomato russet mite population collected in summer 2008 from a greenhouse in the Westland area (The Netherlands) was supplied by Koppert Biological System (Berkel en Rodenrijs, The Netherlands). They were reared in insect cages (Bug-Dorm-44590DH; MegaView Science, Taichung, Taiwan) on 3- to 5-week-old tomato plants (cv. Castelmart), and maintained in a climate room at 25 °C under a 16 h light /8 h dark regime at 60% relative humidity (RH). TRM was not habituated to Moneymaker before being used on the experiments.

### Drought stress regime

Drought stress was attained using the protocol described by Ximénez-Embún et al. (2016) with minor modifications. In brief, tomato plants were maintained well-watered in the greenhouse as described in the previous section until they had developed three expanded leaves (in about 3 weeks). Then plants were transferred to a climate room (same conditions as above) and were randomly divided in two groups: one group for the control and one group for the moderate drought stress treatment. Control plants were watered every 2–3 days to maintain the soil volumetric water content (θ) up to 74%. For moderate stress, watering was stopped for 7 days and thereafter plants were watered to maintain θ between 21 and 30%. The wilting point was avoided as it happens at θ = 16%. θ was determined gravimetrically by recording the pot weight of each plant pot.

The severity of drought stress was assessed by measuring the stomatal conductance (gs) of the sub-terminal leaflet of the third leaf using a leaf porometer (SC-1 Decagon-T, Pullman, WA, USA). Plant growth was estimated by measuring the stem length (distance between the soil and the terminal bud) (Tapia et al. 2016).

### Bioassays

Two experiments were carried out: the first to measure the effect of drought on mite population growth, the second to obtain plant material for the various metabolite analyses. In addition, in the second experiment the plant damage was evaluated. Both experiments were carried out in a climate room under the same environmental conditions as used for the mite rearing.

#### Mite population growth

Tomato plants were assigned to four different groups: two served as controls and two were used for the drought stress treatment. For each, one group was sampled at 7 and the other at 14 days post infestation (dpi). When drought stress conditions had stabilized (at about 7–9 days after stopping irrigation for Moneymaker in our experimental conditions, see Ximénez-Embún et al. 2016; this moment coincided with the plants having four expanded leaves), 12 plants per group were infested with TRM by placing 20 individual adult mites on leaves 2, 3 and 4 using a fine-bristle paint brush. Thus, plants were inoculated with a total 60 TRM each. A thin barrier of Lanolin (Sigma-Aldrich Chemie, Zwijndrecht, The Netherlands) was prepared, using a needleless syringe, around the petioles of the leaf to prevent the mites from escaping. The TRM population density was assessed at the two time points using the protocol described by Glas et al. (2014). In brief, the three infested leaves of each plant were detached and washed one by one for 20 s in a single volume of 25 ml of ethanol 100%. TRM were counted by running 2 ml of leaf washes through a particle count system (PAMAS SVSS, PAMAS, Rutesheim, Germany). The number of particles measured was in the range of 50–200 μm as TRM adult size is ca. 120–150 µm.

#### Plant sampling and plant damage evaluation

In addition to the population growth experiments we performed trials to harvest leaves for analyze plant nutritional composition and defense response. Furthermore, in this set of experiment the plant damage was evaluated. Tomato plants were divided in four different groups combining two treatments: drought stress (control or drought) and TRM infestation (infested or non-infested). When drought stress conditions had stabilized (see ‘Mite population growth’ section), plants were infested with TRM by placing a small piece of an infested leaf (ca. 0.5 cm^2^) on each of the two subterminal leaflets on leaves 2, 3 and 4, resulting in six pieces per plant (Stout et al. 1996). These pieces were cut from highly infested tomato plants and each piece contained around 200–250 mobile stages as determined by stereomicroscope (1200–1500 mites per plant). A thin barrier of Lanolin was prepared, using a needleless syringe, around the petioles of the leaf to prevent the mites from escaping. Seven days after infestation, plant material was collected by pooling the subterminal leaflets of leaves 2, 3 and 4 of each plant. Samples were flash frozen in liquid nitrogen, then ground in liquid nitrogen using a mortar and pestle to a fine powder and stored at − 72 °C. Six plants were sampled for each of the four treatments, except for phytohormone analysis for which 11 plants were sampled per treatment.

To determine the damage produced by TRM on tomato, a plant injury index was established: 0 (healthy leaf), 1 (20% of infected leaflet yellow), 2 (infected leaflet and part of other yellow), 3 (a leaflet dead and more than one leaflet yellow), 4 (two or three leaflets dead) and 5 (dead leaf) as shown in Supplementary Fig. S1. The plant injury index was assessed at 7 and 10 dpi and was averaged for three infested leaves.

### Chemical and biochemical analysis

#### Chemicals and equipment

Unless specified otherwise, all chemical compounds were obtained from Sigma-Aldrich (St Louis, MO, USA). Fluorimetric measurements were made using a Varioskan Flash reader (ThermoFisher Scientific, Wilmington, DE, USA), and spectrophotometric measurements with a VERSAmaxmicroplate reader (Molecular Devices, Sunnyvale, CA, USA).

#### Plant nutritional composition analysis

##### Free sugars

Samples of 40 mg of frozen leaf powder were dried in an oven at 70 °C for 3 days and 2.5 mg of the resulting material was homogenized in 650 µl of ethanol 95% (v/v), heated at 80 °C for 20 min, centrifuged at 10,000 rpm for 10 min, and the supernatant collected. The process was repeated two more times and the three supernatants were pooled. A volume of 750 µl of the mixture was dried on a SpeedVac Concentrator Savant SVC-100H (ThermoFisher Scientific) and redissolved in 500 µl of water. Soluble carbohydrate concentration was estimated by the anthrone method (Maness 2010) using glucose as a standard. In brief, 1 ml of anthrone reagent (0.2% v/v anthrone on 95% sulfuric acid) was added to the extract, heated to 90 °C for 15 min, and the absorbance measured at 630 nm.

##### Free amino acids

The extraction of the free amino acids was done as described by Hacham et al. (2002). Samples of 50 mg of frozen leaf powder were homogenized with 600 µl of water:chloroform:methanol (3:5:12 v/v/v). After centrifugation at 12,000 rpm for 2 min, the supernatant was collected and the residue was re-extracted with 600 µl of the same mixture, pooling the two supernatants. A mixture of 300 µl of chloroform and 450 µl of water were added to the supernatants, and after centrifugation the upper water:methanol phase was collected and dried in the SpeedVac. The samples were dissolved on 100 µl of sodium citrate loading buffer pH 2.2 (Biochrom, Holliston, MA, USA) and 10 µl were injected on a Biochrom 30 Amino Acid Analyser at the Protein Chemistry Service at CIB (CSIC, Madrid, Spain).

##### Soluble protein

Samples of 100 mg of leaf frozen powder were homogenized in 500 µl of 0.15 M NaCl, ground with fine sand. The homogenate was centrifuged at 12,000 rpm for 5 min at 4 °C, and the soluble protein quantified by absorbance at 280 nm on a Nanodrop 1000 spectophotometer (ThermoFisher Scientific).

#### Plant defense proteins

Samples of 100 mg of leaf frozen powder were homogenized with 500 µl of extraction buffer (0.15 M NaCl for protease inhibitors, and 0.1 M phosphate buffer, pH 7.0; 5% w:v polyvinylpolypyrrolidine for oxidative enzymes) and soluble protein quantified as explained above.

##### Protease inhibitors

The inhibitory activity of plant protein extracts was tested against commercial enzymes: papain (EC 3.4.22.2), cathepsin B from bovine spleen (EC 3.4.22.1), trypsin from bovine pancreas (EC 3.4.21.4), α-chymotrypsin from bovine pancreas (EC 3.4.21.1), cathepsin D from bovine spleen (EC 3.4.23.5) and leucine aminopeptidase from porcine pancreas (EC 3.4.11.1), as described by Ximénez-Embún et al. (2016). In brief, samples of 20 µg of plant protein extracts (40 µg in case of leucine aminopeptidase inhibition assay) were preincubated for 10 min with 100 ng of the commercial enzyme, subsequently substrate is added and incubated for a specific time and absorbance is measured. Reaction conditions are summarized in Supplementary Table S1. Results were expressed as a percentage of protease activity inhibited.

##### Oxidative enzymes

Polyphenol oxidase (PPO) activity was analyzed by incubating 20 µl of enzyme extract with cathecol (40 mM final concentration) in 160 µl of Tris-HCl pH 8.5 buffer at 30 °C for 1 h. Absorbance was read at 420 nm. Peroxidase (POD) activity was determined incubating 20 µl of a 1:10 dilution of the enzyme extract with guaiacol (5 mM final concentration) and H_2_O_2_ (2.5 mM final concentration) in 150 µl of potassium phosphate pH 6 buffer at 30 °C for 10 min. Absorbance was read at 470 nm. PPO and POD activities were expressed as nmol substrate metabolized relative to time and total protein content.

### Quantification of phytohormones by means of LC–MS

Phytohormones were extracted adapting the procedure described by Alba et al. (2015). In brief 100 mg of frozen leaf powder was homogenized using a GenoGrinder Precellys24 Tissue Homogenizer (Bertin Technologies, Aix-en-Provence, France) in 1 ml of ethyl acetate spiked with 100 ng of D_6_-SA and D_5_-JA (C/D/N Isotopes, Pointe-Claire, Quebec, Canada) as internal standards. After centrifugation for 20 min at 13,000 rpm and 4 °C, the supernatant was collected and the residue was re-extracted with 0.5 ml of ethyl acetate without internal standards. After centrifugation the supernatant was combined with the previous one and evaporated on a vacuum CentriVap Centrifugal Concentrator (Labconco, Kansas City, MO, USA) at 30 °C. The pellet was re-suspended in 250 µL of 70% LCMS-grade methanol (v/v), centrifuged for 10 min and transferred to liquid chromatography–mass spectrometry (LC–MS) vials (Fisher Scientific, Hampton, NH, USA). A serial dilution of pure standards of OPDA, JA, JA-Ile, SA and ABA was run separately. Phytohormone measurements were conducted on a liquid chromatography tandem mass spectrometry system (Varian 320 Triple Quad LC/MS/MS, Agilent Technologies, Santa Clara, CA, USA). The mobile phase comprised solvent A (0.05% formic acid in water; Sigma-Aldrich) and solvent B (0.05% formic acid in methanol; Sigma-Aldrich). The program was set as follows: 95% solvent A for 1.5 min, followed by 6 min in which solvent B increased till 98% which continued for 5 min, subsequently returning to 95% solvent A for 1 min until the end of the run (18 min in total). The flow was 0.2 ml/min during the whole run. Compounds were detected in the electrospray ionization negative mode. The parent and daughter ions used for these analyses are listed in Supplementary Table S2. For all oxylipins and ABA we used D_5_-JA to estimate the recovery rate and D_6_-SA for SA. The *in planta* hormone concentrations were subsequently quantified using the external standard series. Phytohormone amounts were expressed as ng per g of fresh leaf material.

### Quantification of gene expression via qRT-PCR

To assess the expression levels of *PPO*-*F*, *JPI*-*21*, *TD2*, *PI*-*IIf* and *PR*-*P6* (Glas et al. 2014; Alba et al. 2015) we performed qRT-PCR. Samples of 100 mg of frozen leaf powder were taken for total RNA extraction using the hot phenol method (Verwoerd et al. 1989). The integrity of RNA was checked on 1% agarose gels and subsequently quantified using a NanoDrop 100 spectrophotometer. DNA was removed with DNAse (Ambion, Huntingdon, UK) according to the manufacturer’s instructions, after which a control PCR was carried out to confirm the absence of genomic DNA contamination. cDNA was synthesized from 2 μg total RNA using a poly(dT) primer and M-MuLV Reverse Transcriptase (Fermentas, St. Leon-Rot, Germany) according to the manufacturer’s instructions. cDNA dilutions (1:10) were used as the template in quantitative reverse-transcriptase PCR (qRT-PCR). Reactions were carried out in a total volume of 20 μl containing 0.25 μM of each primer, 0.1 μl ROX reference dye and 1 μl of cDNA template. Two technical replicates were performed per measurement. qRT-PCR was performed with Platim SYBR Green qPCR Super Mix (Invitrogen, Paisley, UK) using an ABI 7500 (Applied Biosystems, Foster City, CA, USA) system. The program was set to 2 min at 50 °C, 10 min at 95°C, 45 cycles of 15 s at 95 °C and 1 min at 60 °C, followed by a melting curve analysis. Target gene expression levels were normalized to those of actin. The normalized relative quantity (NRQ) data were calculated by the ΔCt method: NRQ = (PE_target_^Ct_target^)/(PE_reference_^Ct_reference^), where PE = primer efficiency, and Ct = cycle threshold. The PEs were determined by fitting a linear regression line on the Ct-values of a standard cDNA dilution series. Specific amplification was ensured by melting curve analyses and generated amplicons were sequenced. The primers we used are listed in Supplementary Table S3.

### Statistical analysis

All plant and mite data were checked for the assumptions of normality and heteroscedasticity, and transformed if necessary. Stem length and stomatal conductance were log_10_(x) transformed and analyzed using a three-way ANOVA (using as fixed factors drought treatment, TRM infestation and time) performing a Bonferroni post hoc test to compare drought-stress treatments within each time. The TRM population size was log_10_(x) transformed and analyzed by a two-way ANOVA (drought treatment, time). The significant differences in leaf damage index in control versus moderate drought plants were determined by the non-parametric Mann–Whitney–Wilcoxon test (U-test). The percentage of protein, free amino acids, free sugars and protease inhibition were arcsine √x transformed, phytohormone data were log_10_(x) transformed and gene-expression data (NRQ values) were ln(x + 1) transformed. These data and the oxidative enzyme activities were analyzed using a two-way ANOVA using as fixed factors drought treatment and TRM infestation. A Newman–Keuls post hoc test was performed to test for differences of means between treatments.

## Results

### Effects of drought on stomatal conductance and tomato plant growth

Two experiments were carried out in parallel to assess the effect of drought on mites and on plant growth parameters. The impact of drought stress on stomatal conductance was similar in the two experiments, and it was between 3× and 8× smaller for stressed plants than for control plants (Supplementary Fig. S2a and Table S4). We observed a reduction in stomatal conductance for the control plants during the course of the experiment, but the effect of drought was maintained. Moderate drought also affected plant growth, as stem length was significantly shorter for stressed plants than for control plants at all measured points (Supplementary Fig. S2b and Table S4). The TRM infestation didn´t induce a significant effect on either stomatal conductance or tomato plant growth (Supplementary Table S4). Therefore the two treatments were pooled (i.e., the infested and uninfested plants) within each experiment to produce the Supplementary Fig. S2.

### Effects of drought on *Aculops lycopersici* population growth and plant damage

**Fig. 1:**
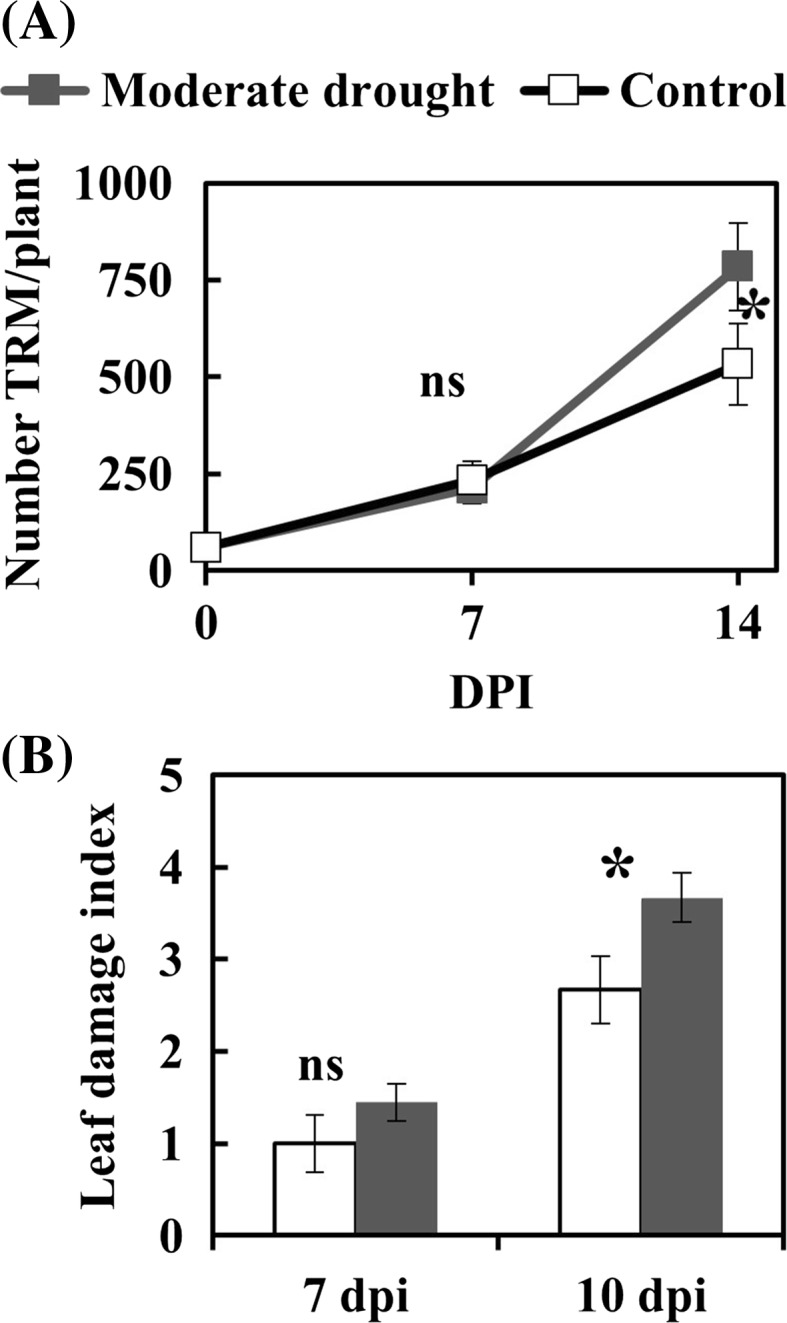
Population growth (**a**) of *Aculops lycopersici* (TRM) and plant injury (**b**) on well-watered (control) and moderately drought stressed tomato plants. Plants were infested with 60 individuals for the population growth assay (**a**) and 1250 individuals for the leaf damage index assay (**b**). Population size was measured at 7 and 14 days post infestation (dpi) while plant damage index was measured at 7 and 10 dpi. Data points represent the mean ± SE. An asterisk indicates significant differences between drought treatments (population assay: two-way ANOVA, Bonferroni post hoc test, *p* < 0.05; Injury index: Mann–Whitney–Wilcoxon test)

When feeding on drought-stressed tomato plants the TRM population grew faster than on control plants (Fig. 1a). However, the difference in population size was significant (F_1,12_ = 5.094, *p* = 0.043) only at 14 dpi. Likewise, plant damage was significantly higher (Mann–Whitney U-test: U = 5.00, Z = − 2.104, *p*(2-tailed) = 0.035) on stressed plants from 10 dpi (Fig. 1b).

### Changes on plant nutritional composition induced by drought and TRM

**Fig. 2:**
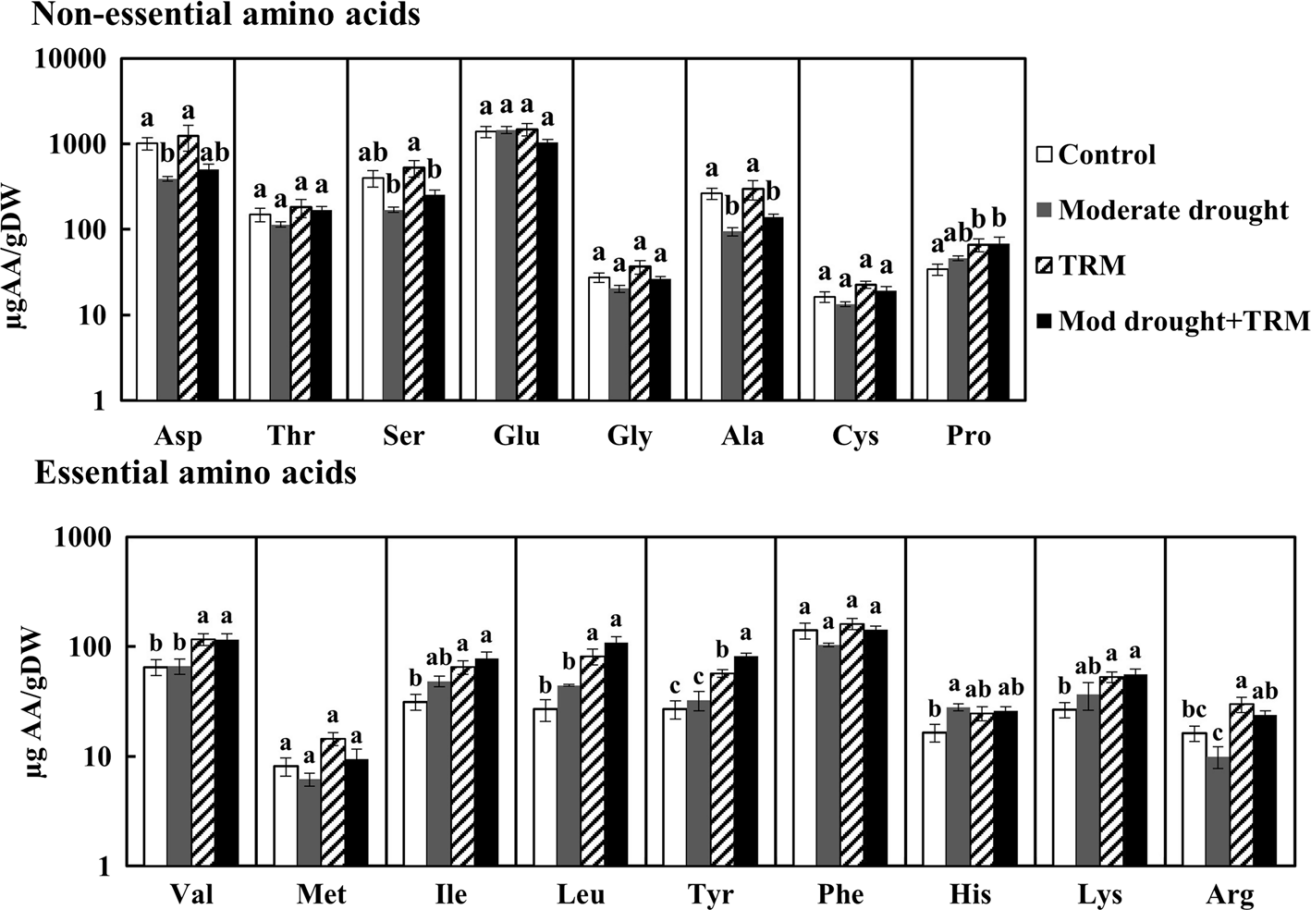
Levels of free amino acids in tomato leaves from control plants and plants under moderate drought stress (Mod drought), and/or infested with *Aculops lycopersici* (TRM) at 7 days post infestation (dpi). The bars represent the mean amount amino acid (µg) per g of dry weight (DW) ± SE represented on a logarithmic scale. The division between essential and non-essential amino acids for *Tetranychus urticae* is based on Rodriguez and Hampton (1966). Different letters indicate significant difference between treatments (two-way ANOVA, Newman–Keuls post hoc test, *p* < 0.05)

The levels of nutrients (protein, free sugars and amino acids) in tomato leaves were affected differently in response to drought stress or TRM infestation (Table 1 and Fig. 2). The results of the two-way ANOVA analysis are presented on the Supplementary Table S5. TRM induced a significant increase of the amount of total protein independent of drought stress. The amino acids analyzed were separated as essential and non-essential ones according to the division made by Rodriguez and Hampton (1966) for *T. urticae*. Moderate drought reduced the amount of the non-essential amino acids aspartic acid (Asp) and alanine (Ala) but increased the amount of the essential histidine (His). Proline (Pro) was the only non-essential amino acid that accumulated to higher levels in response to both TRM infestation and TRM combined with drought stress, whereas alanine levels were also reduced when both stresses were combined. In contrast, TRM alone and combined with drought stress induced the essential ones, valine (Val), isoleucine (Ile), tyrosine (Tyr), lysine (Lys) and leucine (Leu), the latter at 4× higher levels than in the control. The essential amino acid arginine (Arg) was induced by TRM but not when combined with drought stress. Alone drought or TRM did not affect free sugar levels but combined they significantly increased these levels.

**Table 1:** Effect of moderate drought and infestation by *Aculops lycopersici* (TRM) on nutritional composition of tomato leaves (mean ± SE of % dry weight)

	Non-infested		TRM infestation	
	Control	Moderate drought	Control	Moderate drought
Protein	17 ± 2a	18 ± 1a	27 ± 2b	27 ± 3b
Free amino acids	0.37 ± 0.05a	0.27 ± 0.02a	0.44 ± 0.09a	0.28 ± 0.03a
Free sugar	4.8 ± 1.0b	5.2 ± 0.5b	3.8 ± 0.2b	8.1 ± 1.1a

Means within a row followed by different letters are significantly different (two-way ANOVA followed by Newman–Keuls test: *p* < 0.05)

### Effect of drought and TRM on tomato plant defense: phytohormones, defense genes and defense proteins

**Fig. 3:**
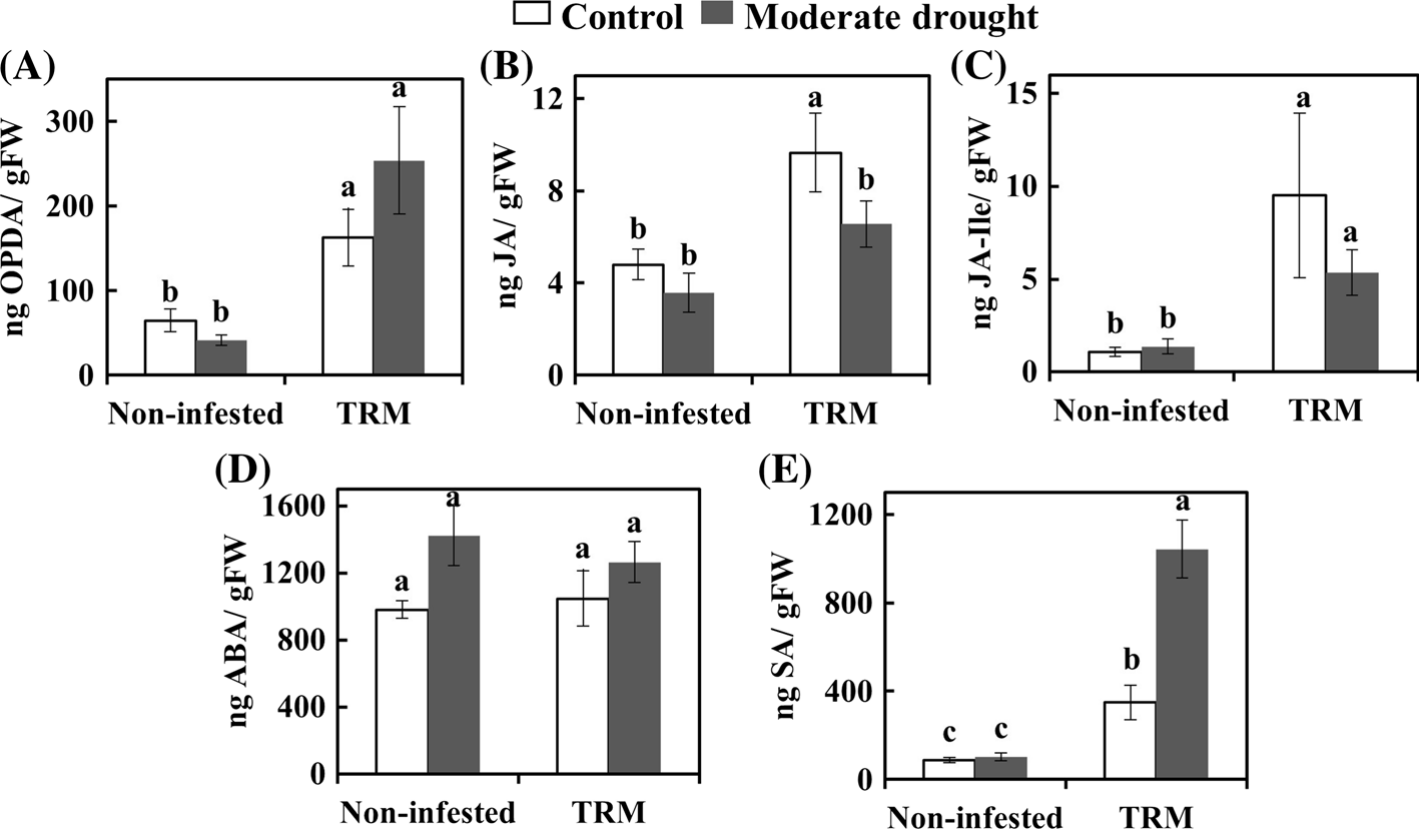
Phytohormone levels in tomato leaves from control plants and plants under moderate drought stress and/or infested with *Aculops lycopersici* (TRM) at 7 days post infestation. The bars represent the mean ng of phytohormone per g of fresh weight (FW) ± SE of endogenous OPDA (**A**), JA (**B**), JA-Ile (**C**), ABA (**D**), SA (**E**). Different letters in each figure indicate significant difference between treatments (two-way ANOVA, Newman–Keuls post hoc test, *p* < 0.05)

**Fig. 4:**
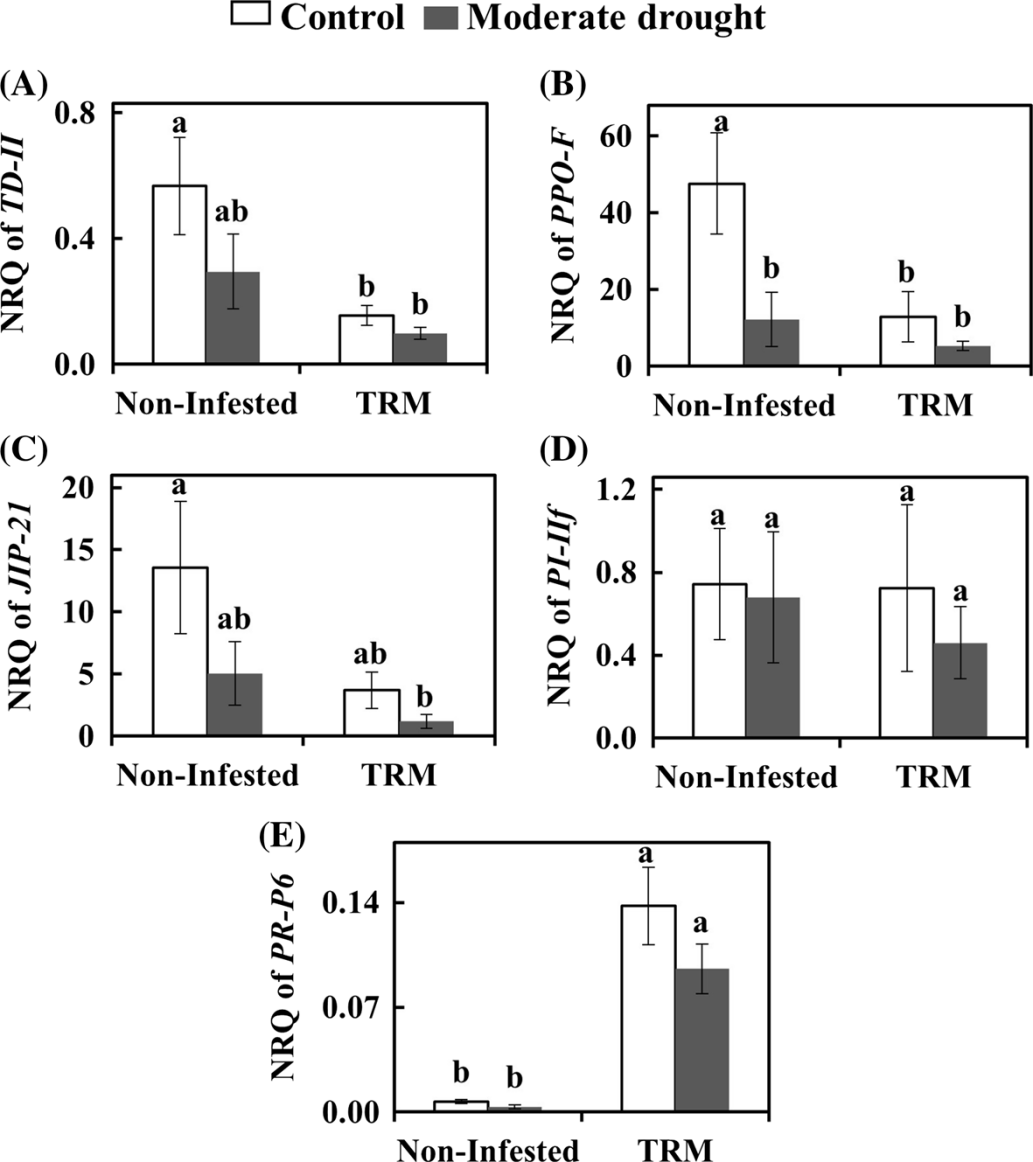
Relative transcript abundance of tomato on control plants and plants under moderate drought stress and/or infested with *Aculops lycopersici* (TRM) at 7 days post infestation. Selected genes mark the JA pathway: *TD*-*II* (**A**), *PPO*-*F* (**B**), *JIP*-*21* (**C**) and *PI*-*IIf* (**D**) and the SA pathway: *PR*-*P6* (**E**). The bars represent the mean normalized relative quantity (NRQ) ± SE. Different letters indicate significant difference between treatments (Two-way ANOVA, Newman–Keuls post hoc test, *p* < 0.05)

The defense response of the plant was analyzed at three levels: phytohormone accumulation (Fig. 3), the transcript levels of marker genes linked to the JA and SA pathways (Fig. 4) and the activity of defense proteins (Table 2). The results of the two-way ANOVA analysis are presented on the Supplementary Table S5.

**Table 2:** Effect of moderate drought and infestation with *Aculops lycopersici* (TRM) on tomato plant defense proteins at 7 days post infestation (mean ± SE)

	Non-infested		TRM infestation	
	Control	Moderate drought	Control	Moderate drought
Cathepsin B	54 ± 2b	47 ± 5b	82 ± 2a	86 ± 2a
Papain	56 ± 3c	57 ± 3c	72 ± 4b	83 ± 2a
Cathepsin D	43 ± 1a	39 ± 3a	40 ± 4a	35 ± 3a
Trypsin	40 ± 4ab	23 ± 5b	50 ± 10a	35 ± 6ab
Chymotrypsin	91 ± 2a	45 ± 12b	95 ± 2a	75 ± 8a
Aminopeptidase	14 ± 3a	10 ± 3a	16 ± 4	19 ± 4
Polyphenol oxidases^1^	5.2 ± 0.5a	4.0 ± 0.6a	7.8 ± 1.3b	4.5 ± 0.4a
Peroxidases^2^	9.4 ± 2.4b	3.9 ± 0.9c	29.9 ± 6.4a	12.1 ± 2.6b

Means within a row followed by different letters are significantly different (Two-way ANOVA, Newman–Keuls post hoc test, *p* < 0.05)

^1^PPO: nmol Cathecol metabolized/mg protein * min

^2^POD: nmol Guaiacol metabolized/mg protein * min

TRM induced a twofold increase in the accumulation of JA, a 2.5-fold increase in OPDA (the precursor of JA) and an 8.7-fold increase in Ja-Ile (the bioactive form of JA) in tomato plants (Fig. 3A–C). In addition, TRM induced a fourfold increase in SA accumulation (Fig. 3E). When combining TRM infestation with drought stress the amount of OPDA increased fourfold and those of SA 12-fold (Fig. 3A, E). However the induction of JA accumulation by TRM was antagonized when combined with drought (Fig. 3B). Moderate drought affected ABA accumulation significantly (two-way ANOVA: *p* = 0.007), but the post hoc analysis did not detect significant differences among treatments (Fig. 3D).

TRM infestation combined or not with drought stress significantly down regulated the expression of the JA-marker genes *TD*-*II* and *PPO*-*F* (Fig. 4A, B), whereas *JIP*-*21* was only down regulated when TRM and drought were combined (Fig. 4C). Moreover, TRM did elevate transcript accumulation for the SA-marker gene *PR*-*P6* more than 20× in both cases: with and without drought (Fig. 4E). Moderate drought, in contrast, down regulated the expression of the JA marker gene*PPO*-*F*, in non-infested plants by fourfold (Fig. 4B).

The defense proteins were divided in two categories: protease inhibitors and oxidative enzymes (Table 2). From the different protease inhibitors, TRM infestation increased the total activity of cysteine protease (cathepsin B and papain) inhibitors in both control and drought treatments. Drought decreased the total inhibitory activity of serine protease (trypsin and chymotrypsin) inhibitors. Cathepsin D and aminopepetidase inhibitory activities were not affected by any of the treatments. The total activity of the oxidative enzymes PPO and POD was significantly increased by TRM. Drought stress decreased the POD activity, but when combined with TRM infestation PPO and POD activities were equal to those of the control.

## Discussion

Our data show that tomatoes infested with TRM increase their levels of total protein and of several free amino acids while promoting SA-responses and upregulating the activity of cysteine protease inhibitors, polyphenol oxidases and peroxidases. Our data also show that drought stress antagonized the accumulation of POD on TRM-infested plants and synergized the accumulation of free sugars and SA. Finally, we demonstrate that this coincided with an increase in TRM population growth and damage on drought-stressed plants. Previously we assessed the nutrient composition and induced defences of normal and drought-stressed tomatoes infested with *T. urticae* or *T. evansi*. All these data together are summarized in Table 3.

**Table 3:** Summary of the effects of *Tetranychus urticae* (Tu), *T. evansi* (Te) and *Aculops lycopersici* (TRM, tomato russet mite) infestation and their combination with drought (Dro) on the levels of tomato plant nutrients and defenses

	Tu^a,b,c^	Te^b,c,d^	TRM^e,f^	Dro + Tu^a^	Dro + Te^d^	Dro + TRM^f^
Nutrients						
Free sugars	↑	↑	0	↑	↑	↑
Protein	0	0	↑	0	↓	↑
Non-essential aa	0	0	0	0	0	0
Essential aa	0	0	↑	↑	↑	↑
Proline	0	0	↑	↑	↑	↑
Defense						
JA	↑	0	↑	na	na	0
JA-associated genes	↑	0^b,c^ ↑^b^	↓	na	na	↓
Cystein PI	0	↑	↑	0	↑	↑
Serine PI	↑	0^d^ ↓^c^	0	↑	0	0
Poliphenol oxidases	0	↑	↑	0	0	0
Peroxidases	0	↑	↑	0	0	0
SA	↑	0	↑	na	na	↑
SA-associated genes	↑	0	↑	na	na	↑

↑ represents an increase, ↓ represents a decrease and 0 indicates absence of effect with respect to the control (well-watered uninfested) plants

*na* not assessed

^a^Ximénez-Embún et al. (2017), ^b^ Alba et al. (2015), ^c^ Sarmento et al. (2011), ^d^ Ximénez-Embún et al. (2016), ^e^ Glas et al. (2014), ^f^ data from this article

We showed that drought-stressed tomato plants reconfigure their metabolism, as previously reported (Bauer et al. 1997; English-Loeb et al. 1997), interfering on TRM-plant in a way that leads to stressed-plant becoming a better host for TRM. Interestingly, drought and TRM synergized accumulation of free sugars which have been shown to act as a phagostimulant to other mite species (Wermelinger et al. 1991; Showler 2013). Also in previous studies we observed that drought-induced accumulation of free sugars and amino acid in tomato plants coincides with improved performance of the herbivorous mites *T. evansi* (Ximénez-Embún et al. 2016) and *T. urticae* (Ximénez-Embún et al. 2017). In contrast, here we did not observed a reduction in soluble protein and an increase in total free amino acids, indicating that there was not a mobilization of protein into free amino acids, as has been observed previously in other plant—spider mite interaction (Ximénez-Embún et al. 2016). Accordingly, the amounts of most amino acids remained unaltered in drought stresses plants, except for a reduction of aspartic acid and alanine contents and an increase of histidine levels. Proline accumulation, an indicator of drought stress (Showler 2013), was not affected by our drought stress treatment. However, proline accounts for only a small fraction of the total concentration of osmotically active solutes in tomato (Pérez-Alfocea et al. 1993) and proline content in tomato leaves has been reported to fluctuate in response to leaf age, and light radiation intensity (Claussen 2005), which may explain the absence of a proline response in this work when compared to previous studies (Ximénez-Embún et al. 2016, 2017). Nevertheless, we observed a reduction in stomatal conductance and in plant growth which clearly indicates that the drought treatment did affect the plants (Harb et al. 2010; Hummel et al. 2010).

The first important aspect that determines plant palatability is its nutritional composition. Reconfiguration of the plant’s primary metabolism during herbivory (Zhou et al. 2015), i.e., mainly that of free carbohydrates and amino acids, has been reported to affect the pool of precursors for defense compounds, the amount of available energy for the plant and may indicate reallocation of nutrients e.g. to storage tissues (Steinbrenner et al. 2011; Zhou et al. 2015). Our data show that TRM infestation alone does not affects the accumulation of free sugars and total free amino acids significantly. In contrast, we observed increased accumulation of soluble protein. Something similar was observed for tomato plants infested with the generalist leaf feeder *Helicoverpa zea* (Steinbrenner et al. 2011). It is not likely that the increase in total soluble protein is primarily due to an increase in defensive enzymes. For example, the concentration of protease inhibitors in plant leaves is low (0.01–0.1%) compared to the total soluble protein (Bolter and Jongsma 1997). The concentrations of several essential amino acids increased during TRM infestation significantly, with isoleucine and leucine levels being elevated the strongest. Possibly this is causally related to the increase in the JA conjugate JA-Ile which plays a decisive role in plant defense signalling (Ataide et al. 2016; Schuman and Baldwin 2016). Proline, a non-essential amino acid, was also induced by TRM reminiscent of *Manduca sexta* on tomato (Gomez et al. 2012) and by the pea aphid on *Medicago truncatula* (Guo et al. 2013). Proline can be used by mosquitoes as a direct energy substrate for glycolysis and ATP production (Scaraffia and Wells 2003) and have shown a phagostimulant effect on *T. evansi* (Ximénez-Embún et al. 2016), it would be interesting to test its effect on TRM population growth.

The second important aspect that determines plant palatability and mite population growth is plant resistance as determined by its defense responses. As mentioned before, we investigated plant defense at three levels: at the phytohormone-level, the marker-gene expression level and at the defense-protein activity level. We found that TRM elevated the accumulation of JA-precursor OPDA, JA, the JA-derivative JA-Ile and of SA, whereas none of these phytohormones was induced by drought. However, in response to TRM the transcript levels of the SA-marker gene (*PR*-*P6*) increased, whereas those of the JA-marker genes, *JIP*-*21*, *PPO*-*F* and *TD*-*II* decreased. This is in line with the results of Glas et al. (2014) who showed that TRM suppresses selectively the expression of JA-dependent defense genes downstream of phytohormone accumulation. The mechanism by which this suppression happens is still unknown, some effectors have been described in other spider mites like *T. urticae* (Villarroel et al. 2016) but not in TRM. The *JIP*-*21* and *PI*-*IIf* genes encode serine protease inhibitors and, accordingly, also the plant’s protease inhibition activity against trypsin and chymotrypsin was not induced by TRM. The expression of these genes is strongly depending on JA (Ament et al. 2004; Li et al. 2004) and can be antagonized or synergized by SA-responses (Mur et al. 2006). However, suppression of *JIP*-*21* and *PI*-*IIf* by TRM was shown not to be due to induced SA-responses (Glas et al. 2014). In contrast, TRM induced an increase in the activities of cysteine (papain and cathepsin B) protease inhibitors (PIs) and of the oxidative enzymes PPO and POD, similar to *T. evansi* (Ximénez-Embún et al. 2016) despite suppression of *PPO*-*F* gene expression (Glas et al. 2014) suggesting other PPO genes to be responsible for this effect (Thipyapong and Steffens 1997; Constabel and Ryan 1998). In addition, also some cysteine protease inhibitors are known to be JA-responsive (Li et al. 2004) suggesting distinct subsets of cysteine PIs may be responsible for this effect reminiscent of these genes in rice (Dutt et al. 2012). So far, the phytophagous mites analysed belonging to the family Tetranychidae (*T. urticae* and *T. evansi)* and Tenuipalpidae (*Brevipalpus chilensis*) rely mainly on cysteine proteases for digestion (Carrillo et al. 2011; Santamaria et al. 2015a; Ximénez-Embún et al. 2016). In addition, cysteine protease inhibitors have been shown to be detrimental to tetranychid mites (Santamaria et al. 2015b). However, it is unknown which type of proteases are involved in the digestion of dietary proteins in TRM and we could not collect sufficient TRM material to perform the analysis. This raises an interesting question: although it was shown that suppression of defenses—i.e., JA- and SA-defenses by *T. evansi* and JA-defenses by *A. lycopersici*—is beneficial for mites and decreases plant resistance, why do all three mite species—including the inducer of JA and SA defenses *T. urticae*—induce accumulation of cysteine protease inhibitors? Hence, it would be interesting to see whether these mites differ in sensitivity to this type of protease inhibitor or whether the role of cysteine protease inhibitors within the collective defense response of the plant is minor. The use of tomato plants with this class of inhibitors silenced could be instrumental for answering this question.

The hormonal responses in plant when experiencing a combination of biotic and abiotic stresses are complex as many of these pathways interact via cross-talk (Thaler and Bostock 2004; Atkinson et al. 2015). The effect size of induction in different tomato leaves, i.e., young versus old, can differ in magnitude although they are all inducible (Stout et al. 1996) and our measurements reflect the average of these responses. Moreover, the accumulation of hormones is dynamic in time and space and the quantities one measures inside of a single leaf or leaflet will depend on several factors such as the time of day (Atamian and Harmer 2016), on where the stress started (i.e., is it a local or a systemic stress?) and on when it started (Eckardt 2015). For instance ABA accumulation typically peaks during the first phase of a drought response but ceases again while the stress continues (Thompson et al. 2007). This might explain why we didn´t observed differences in ABA accumulation between treatments on tomato at 7 dpi. Other phytohormones involved in the early drought response of tomato are JA and SA (Muñoz-Espinoza et al. 2015), though there is not much information on their role in the later phases of the response. In our experiments, drought stress didn´t induce JA or SA. However, when drought stressed plants were also infested with TRM the accumulation of SA increased to almost threefold compared to the levels induced by the mite on control plants. This synergistic effect may be caused by hormonal crosstalk between JA and SA (Howe and Jander 2008; Schuman and Baldwin 2016) since the combination of TRM and drought reduces the levels of JA with a similar trend for JA-Ile. However, Glas et al. (2014) showed that suppression of JA-defense by TRM occurres independent from hormonal crosstalk. Although the effect of drought stress alone on JA and JA-Ile was not significant, it decreased the transcript levels of the JA-marker gene *PPO*-*F*, and reduced the activity of serine protease (trypsin and chymotrypsin) inhibition, and of POD. This indicates that the inducibility of JA-related responses by herbivores is strongly reduced in drought stressed tomatoes.

This leads us to suggest that, under our conditions, the reduction of tomato defenses due to drought stress contributes to the increase in the plant’s palatability to TRM. This implies that for TRM effects of drought on defenses may play a bigger role in promoting the mite’s population growth-response than for the spider mite *T. evansi* for which especially changes in nutritional quality were associated with an increase in the mite’s performance (Ximénez-Embún et al. 2016). However, in literature results on the effect of drought stress on tomato plant defenses are not always consistent (English-Loeb et al. 1997), possibly as a consequence of the spatiotemporal dynamics of (cross-talking) hormonal responses in relation to differences between sampling protocols and experimental conditions. Therefore, the most important readout is the performance of the herbivore since clearly not only spider mites (Ximénez-Embún et al. 2016, 2017) but also russet mites benefit from drought and become a more aggressive pest on tomato although the plant-physiological basis for these effects can be different. Such increased aggressiveness not only decreases the response time of growers but also hampers the already troublesome biological control of russet mites even further (Duso et al. 2010; van Houten et al. 2013). Besides, our results provide an experimental framework for screening for drought-resistant tomato accessions that will be at the same time resistant to herbivore mites to ensure crop protection also under a changing climate in times that pesticides are increasingly banned from the production process.

## Conclusions

Our data indicate that the joint action of both drought stress and tomato russet mite infestation can synergize each other’s effects on the plant’s nutritional quality. These effects are characterized by a disproportional increase in the plant’s levels of free sugars, and some essential amino acids. Moreover, TRM-infested plants exposed to drought exhibit a weakened JA-response paralleled by an amplified SA-response. This indicates that tomatoes grown under a dry regime or in areas where periods of drought are expected to increase in number and length, TRM may become an increasingly problematic pest and under the influence of climate change this will especially account for tomatoes grown in the open-field. Also other mite pests perform better on drought-stressed plants (Ximénez-Embún et al. 2016, 2017) and may become more serious pests in the future. To be ahead of this danger we should search for resistance traits that are displayed equally strong, or stronger, during drought, for reminiscent of, for example, the efforts for increasing diseases resistance together with drought tolerance in rice (Wang et al. 2017), to protect tomato cultivation in the future.

## Electronic supplementary material

Below is the link to the electronic supplementary material.

Supplementary material 1 (PDF 640 kb)
