# Supplementary material for: Drought stress promotes the colonization success of a herbivorous mite that manipulates plant defenses
Source: Exp Appl Acarol. 2017 Nov 29;73(3):297–315. doi: 10.1007/s10493-017-0200-4 (PMC5727147; doi:10.1007/s10493-017-0200-4)

Drought stress promotes the colonization success of a herbivorous mite that manipulates plant defences

Miguel G. Ximénez-Embún, Joris J. Glas, Felix Ortego, Juan M. Alba , Pedro Castañera , Merijn R. Kant

Supplementary data

Supplementary Table S1. Summary of analytical methods to assess the inhibitory activity of plant protein extracts [1]

Commercial enzyme [2]	Substrate [3]	Buffer [4]	Incubation	Measurement [5]
Cathepsin B from bovine spleen (EC 3.4.22.1)	Z-RR-AMC	100 mM NA phosphate, pH 6.0 (10 mM L-cysteine, 10 mM EDTA, 0.01% (v/v) Brij 35)	1 h at 28 ºC	excitation filter 350 nm emission filter 465 nm
Papain from *Carica papaya* (EC 3.4.22.2)	Z-FR-AMC	100 mM Na phosphate, pH 6.0 (10 mM L-cysteine, 10 mM EDTA, 0.01% (v/v) Brij 35)	1 h at 28 ºC	excitation filter 350 nm emission filter 465 nm
Cathepsin D from bovine spleen (EC 3.4.23.5)	MocAc-GKPILFFRLK (Dnp)-D-R-NH2	100 mM sodium citrate, pH 3.5 (0.15M NaCl, 5 mM MgCl2)	10 min at 30 ºC	excitation filter 328 nm emission filter 393 nm
Trypsin from bovine pancreas (EC 3.4.21.4),	Z-LA-AMC	100 mM Tris-HCl, pH 7.5 (0.15M NaCl, 5 mM MgCl2)	1 h at 35 ºC	excitation filter 350 nm emission filter 465 nm
α-Chymotrypsin from bovine pancreas (EC 3.4.21.1),	SucAAPF-AMC	100 mM Tris-HCl, pH 7.5 (0.15M NaCl, 5 mM MgCl2)	1 h at 35 ºC	excitation filter 350 nm emission filter 465 nm
Leucine aminopeptidase from porcine pancreas (EC 3.4.11.1).	LpNa	100 mM Tris-HCl, pH 8 (0.15M NaCl, 5 mM MgCl2)	1 h at 30 ºC	absorbance at 410 nm

[1] Procedures adapted from Ximénez-Embún et al. (2016). Samples of 20 µg of plant protein extracts (40 µg in case of leucine aminopeptidase) were preincubated for 10 min with 100 ng of the commercial enzyme.

[2] All purchased from Sigma-Aldricht (St Luis, USA).

[3] The substrates were added at a final concentration of 20 µM. Z-RR-AMC (N-carbobenzoxyloxy-Arg-Arg-7-amido-4-methylcoumarin) for cathepsin B, Z-FR-AMC (N-carbobenzoxyloxy-Phe-Arg-7-amido-4-methylcoumarin) for papain, Z-LA-AMC (Z-L-Arg-7-amido-4-methylcoumarin) for trypsin, SucAAPF-AMC (Suc-Ala-Ala-Pro-Phe-7-amido-4-methylcoumarin) for chymotrypsin, all purchased from Calbiochem (MerkMilipore, Billerica, USA), MocAc-GKPILFFRLK(Dnp)-D-R-NH2 from Peptanova (Germany) for cathepsin D, and LpNa (L-leucine p-nitroanilide) from Sigma-Aldricht (St Luis, USA) for leucine aminopeptidase.

[4] Concentrations are expressed at molarity in the reaction mixture.

[5] AMC (7-amino-4-methylcoumarin) (Bachem, Swizerland) as standard for all fluorescent substrates, except MCA (MoCAC-Pro-Leu-Gly) (Peptanova GmbH, Germany) for cathepsin D. Double blanks were used to account for spontaneous breakdown of substrates and the plant protease activity, and all assays were done in duplicate.

Supplementary Table S2 Parameters used for detection of phytohormones and related compounds by LC-MS/MS

Compound	Molecular ion [M-H] (*m/z*)	Fragment ion (*m/z*)
**OPDA**	291	165
**JA**	209	59
**D_5-JA (Internal Standard)**	213	61
**JA-Ile**	322	130
**SA**	137	93
**D_6-SA (Internal Standard)**	141	97
**ABA**	263	153

Supplementary Table S3 Nucleotide sequence of primers used for qRT-PCR analysis

Target Gene	Name	GenBank (GB) accession	Gen Model ITAG2.3:	Forward Primer 5' → 3'	Reverse Primer 5' → 3'
PPO-F	*Polyphenol-oxidase-F*	AK247126.1	Solyc08g074630.1.1	CGGAGTTTGCAGGGAGTTATAC	TTGATCTCCACACTTTCAATGG
JIP-21	*Jasmonate-inducible protein 21*	AJ295638.1	Solyc03g098790.1.1	ACTCGTCCTGTGCTTTGTCC	CCCAAGAGGATTTTCGTTGA
TD2	*Threonine Deaminase-2*	M61915.1	Solyc09g008670.2.1	TGCCGTTAAAAATGTCACCA	ACTGGCGATGCCAAAATATC
PI-IIf	*Proteinase Inhibitor IIf*	AY129402.1	Solyc03g020080.2.1	GACAAGGTACTAGTAATCAATTATCC	GGGCATATCCCGAACCCAAGA
PR-P6	*Pathogenesis-related protein P6*	M69248.1	Solyc00g174340.1.1	GTACTGCATCTTCTTGTTTCCA	TAGATAAGTGCTTGATGTCCA
Actin	*Actin*	XM_004235020.1	Solyc03g078400.2.1	TTAGCACCTTCCAGCAGATGT	AACAGACAGGACACTCGCACT

Supplementary Table S4 Results of the three-way ANOVA analysis.

	Drought			TRM			Time			Drought*TRM[a]		
	**F**	**df**	***p***	**F**	**df**	***p***	**F**	**df**	***p***	**F**	**df**	***p***
**Mite population growth experiment**												
**Stomatal conductance**	35.6	1,57	**<0.001**	4.09	1,57	0.06	421	2,57	**<0.001**	0.01	1,57	0.947
**Stem length**	10.6	1,57	**<0.001**	0.023	1,57	0.876	12.9	1,57	**<0.001**	0.31	1,57	0.578
**Plant sampling and plant damage evaluation experiment**												
**Stomatal conductance**	4.42	1,55	**<0.001**	0.965	1,55	0.387	18.1	1,55	**<0.001**	2.10	1,55	0.153
**Stem length**	193	1,55	**<0.001**	1.382	1,55	0.246	290	1,55	**<0.001**	8.30	1,55	**0.006**

[a]From the different possible interactions between factors only the one studied is presented

Supplementary Figure S5 Results of the two-way ANOVA analysis.

	Moderate drought			TRM			Drought*TRM		
	**F**	**df**	***p***	**F**	**df**	***p***	**F**	**df**	***p***
**Nutrients**									
**Free sugars**	10.35	1,20	**0.004**	1.275	1,20	0.272	5.915	1,20	**0.024**
**Protein**	0.139	1,20	0.714	20.47	1,20	>**0.001**	0.148	1,20	0.705
**Total free aa**	5.074	1,20	**0.036**	0.66	1,20	0.426	0.229	1,20	0.642
**Non-essential amino acids**									
**Asp**	13.020	1,20	**0.002**	0.450	1,20	0.510	0.006	1,20	0.940
**Thr**	0.461	1,20	0.505	2.676	1,20	0.117	0.293	1,20	0.594
**Ser**	12.927	1,20	**0.002**	2.510	1,20	0.129	0.004	1,20	0.949
**Glu**	0.839	1,20	0.371	0.927	1,20	0.347	1.965	1,20	0.176
**Gly**	4.041	1,20	0.058	3.643	1,20	0.071	0.024	1,20	0.878
**Ala**	19.703	1,20	**0.000**	1.274	1,20	0.272	0.354	1,20	0.558
**Cys**	1.978	1,20	0.175	9.331	1,20	**0.006**	0.008	1,20	0.929
**Pro**	0.976	1,20	0.335	9.218	1,20	**0.007**	0.524	1,20	0.477
**Essential amino acids**									
**Val**	0.022	1,20	0.882	18.315	1,20	**0.000**	0.04	1,20	0.844
**Met**	3.219	1,20	0.088	3.201	1,20	0.089	0.924	1,20	0.348
**Ile**	3.555	1,20	0.074	14.763	1,20	**0.001**	0.372	1,20	0.549
**Leu**	6.472	1,20	**0.019**	39.416	1,20	**0.000**	0.038	1,20	0.848
**Tyr**	9.716	1,20	**0.005**	71.310	1,20	**0.000**	1.599	1,20	0.221
**Phe**	2.436	1,20	0.134	3.545	1,20	0.074	0.305	1,20	0.587
**His**	5.342	1,20	**0.032**	1.289	1,20	0.270	3.358	1,20	0.082
**lys**	1.683	1,20	0.209	17.287	1,20	**0.000**	0.71	1,20	0.410
**Arg**	4.809	1,20	**0.040**	26.030	1,20	**0.000**	0.24	1,20	0.630
**Phytohormones**									
**OPDA**	0.026	1,39	0.871	36	1,39	**<0.001**	3.560	1,39	0.067
**JA**	6.02	1,39	**0.019**	9.812	1,39	**0.003**	0.219	1,39	0.642
**JA-Ile**	0.955	1,39	0.334	10.76	1,39	**0.002**	0.565	1,39	0.457
**ABA**	8.04	1,39	**0.007**	0.195	1,39	0.661	0.228	1,39	0.636
**SA**	12.48	1,39	**0.001**	95.88	1,39	**<0.001**	8.941	1,39	**0.005**
**Gene expresion**									
TD-II	3.330	1,16	0.087	11.14	1,16	**0.004**	1.160	1,16	0.297
PPO-F	7.566	1,15	**0.015**	4.965	1,15	**0.042**	1.887	1,15	0.120
JIP-21	5.200	1,16	**0.036**	5.203	1,16	**0.036**	0.288	1,16	0.599
PI-IIf	0.208	1,16	0.654	0.273	1,16	0.608	0.005	1,16	0.943
PR-P6	1.543	1,16	0.232	41.24	1,16	**<0.001**	1.054	1,16	0.320
**Defense proteins**									
**Cathepsin B**	0.209	1,20	0.653	144.1	1,20	**<0.001**	4.386	1,20	0.0499
**Papain**	3.481	1,20	0.0776	42.3	1,20	**<0.001**	2.632	1,20	0.1212
**Cathepsin D**	1.708	1,20	0.2069	1.1	1,20	0.302	0.104	1,20	0.7507
**Trypsin**	5.673	1,20	**0.028**	2.8	1,20	0.113	0.021	1,20	0.8873
**Chymotrypsin**	23.54	1,20	**<0.001**	6.1	1,20	**0.023**	1.904	1,20	0.1836
**Aminopeptidase**	0.059	1,20	0.8114	2.3	1,20	0.149	1.398	1,20	0.2517
**Polyphenol oxidases**	8.393	1,20	**0.009**	3.756	1,20	0.0676	0.664	1,20	0.4254
**Peroxidases**	16.46	1,20	**<0.001**	29.38	1,20	**<0.001**	0.014	1,20	0.9082
	**F**	**df**	***p***	**F**	**df**	***p***	**F**	**df**	***p***
	**Drought**			**TRM**			**Drought*TRM**		

Supplementary Figure S1 Leaf damage index explanation

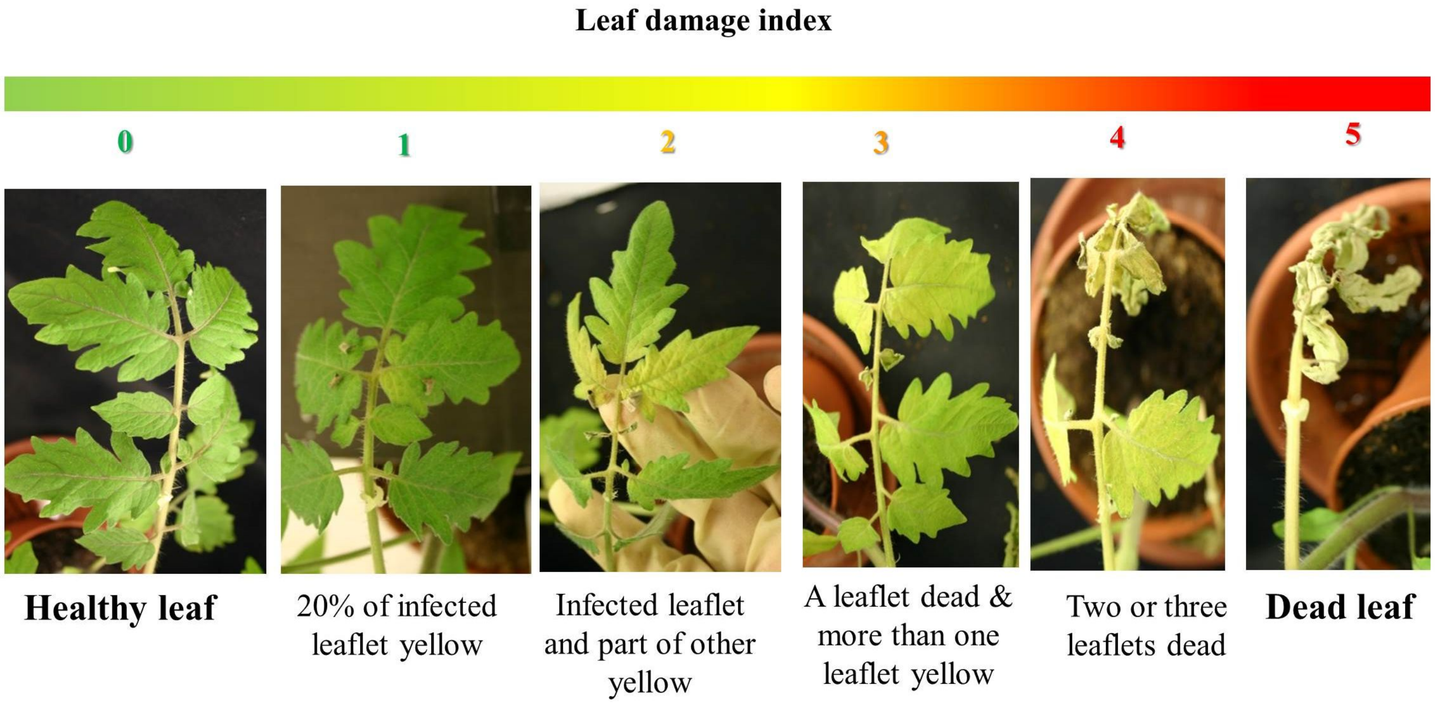

Supplementary Figure S2 Effect of moderate drought on A) tomato stomatal conductance (gs) and B) stem length at mite infestation (mi) at 7 and 14 days post infestation (dpi). Data (mean ± SE) are average of the values on infested and non-infested w.t. plants on TRM population growth and plant material experiments, as mite infestation didn´t show a significant effect. An asterisk indicates a significant difference between drought and control treatments at each time (Three-way ANOVA, Bonferroni *post hoc* test, $p<0.05$).

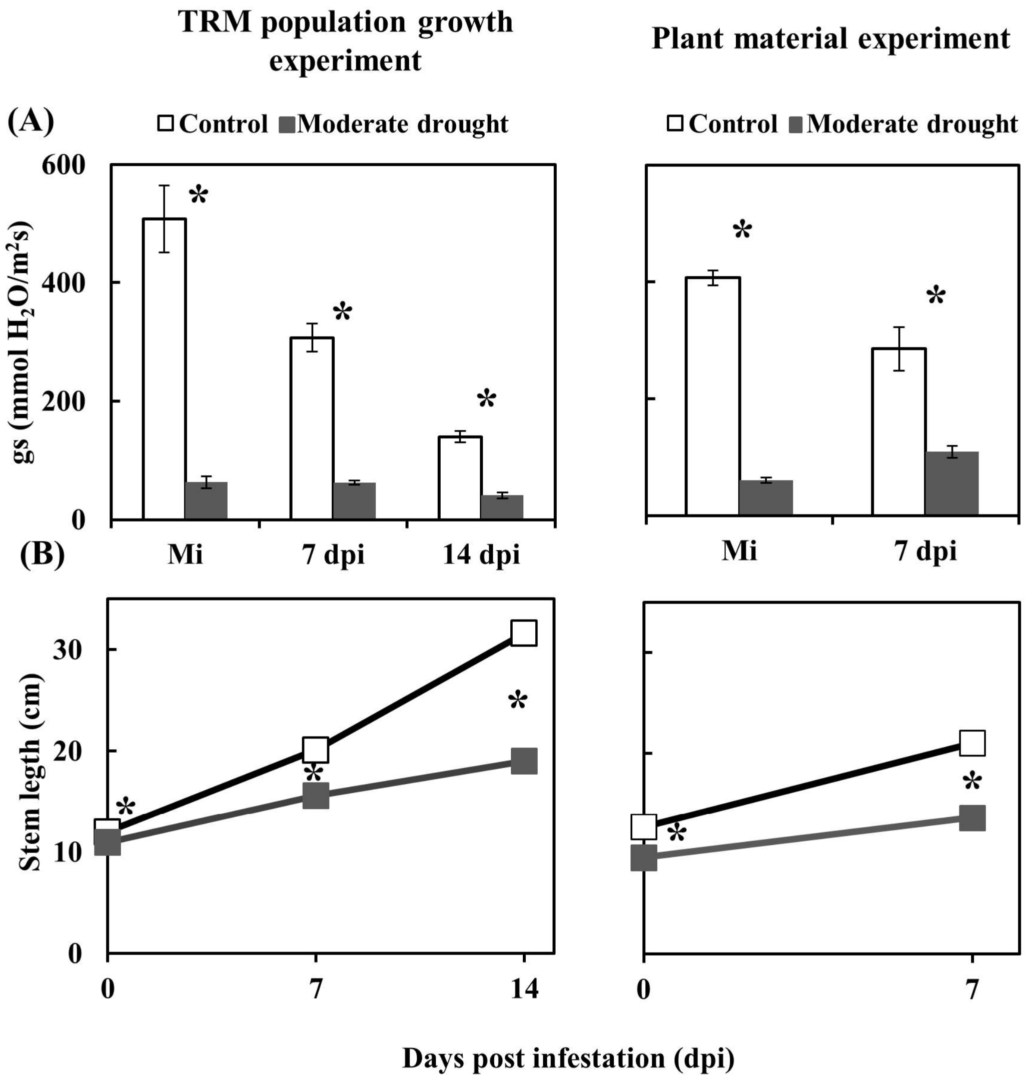

Supplement: Supplementary file 1 — Supplementary material 1 (PDF 640 kb) [file 10493_2017_200_MOESM1_ESM.pdf]
